# Ocurrence of rotavirus and picobirnavirus in wild and exotic avian from amazon forest

**DOI:** 10.1371/journal.pntd.0008792

**Published:** 2021-09-10

**Authors:** José Wandilson Barboza Duarte Júnior, Elaine Hellen Nunes Chagas, Ana Carolina Silva Serra, Lizandra Caroline dos Santos Souto, Edvaldo Tavares da Penha Júnior, Renato da Silva Bandeira, Ricardo José de Paula Souza e Guimarães, Hanna Gabriela da Silva Oliveira, Thaymis Kiara Santos Sousa, Cinthia Távora de Albuquerque Lopes, Sheyla Farhayldes Souza Domingues, Helder Henrique Costa Pinheiro, Yashpal Singh Malik, Felipe Masiero Salvarani, Joana D’Arc Pereira Mascarenhas

**Affiliations:** 1 Evandro Chagas Institute, Ministry of Health, Ananindeua, Pará, Brazil; 2 University of the State of Pará, Institute of Veterinary Medicine, Castanhal, Pará, Brazil; 3 Federal University of Pará, Belém, Pará; 4 Indian Veterinary Research Institute, Izatnagar, Bareilly, India; WRAIR, UNITED STATES

## Abstract

The present study reports the occurrence of rotavirus A (RVA), rotavirus D (RVD), rotavirus F (RVF), rotavirus G (RVG), and picobirnavirus (PBV) in fecal specimens of wild (n = 22), and exotic birds (n = 1) from different cities of Pará state. These animals were hospitalized at Veterinary Hospital of the Federal University of Pará, Brazil, in a period from January 2018 to June 2019. The animals exhibited different clinical signs, such as diarrhea, malnutrition, dehydration, and fractures. The results showed 39.1% (9/23) of positivity for RVA by RT-qPCR. Among these, one sample (1/9) for the NSP3 gene of T2 genotype was characterized. About 88.9% (8/9) for the VP7 gene belonging to G1, G3 equine like and G6 genotypes, and 55.5% (5/9) for the VP4 gene of **P[2]** genotype were obtained. In the current study, approximately 4.5% of the samples (1/23) revealed coinfection for the RVA, RVD and RVF groups. Furthermore, picobirnavirus (PBV) was detected in one of the 23 samples tested, and was classified in the Genogroup I. The findings represent the first report of RVA, RVD, RVF, RVG, and PBV genotypes in wild birds in Brazil, and due to wide distribution it can implies potential impacts of RVs, and PBVs on avian health, and other animals contributing to construction of new knowledge, and care perspectives.

## Author summary

The anthropic action in the Amazon region directly influences the relationship among pathogens, and animal hosts. This kind of action could move different species of animals from their natural habitat to urban or rural areas, which can lead to the dispersion of different etiological agents such rotavirus and picobirnavirus, showing their zoonotic potential, and increasing their genetic diversity. This study detected the circulation of rotavirus in 9/23 samples of wild, and exotic avian, and 1/23 for picobirnavirus from different cities of Pará state, Brazil. We report the first occurrence of rotavirus A, D, F, G, and Genogroup I of picobirnavirus in wild, exotic birds in Brazil, and worldwide. Our data correlated the pathogens with cities, and symptoms. The data emphasize the scarce of epidemiological monitoring of these pathogens, and the importance of epidemiological studies to obtain the ecology, and evolution of rotavirus, and picobirnavirus in animals population as well as the negative impacts in animal health.

## Introduction

The Brazilian Amazon biome is the largest ecosystem of wildlife biodiversity, and Brazil is the third country that protects the biggest diversity of birds in the world, with registration of 1.919 species [1], and global distribution of 10.429 bird species [2]. Pará is the second state with the extensive territorial extension covering the Amazon biome. However, it is also a region that suffers anthropic pressure with the advance of deforestation, fires, hunting, and illegal sale of both wild, and exotic species. This situation favors the proximity of wildlife with other animals, and humans, which can lead to maximization, and dispersion of zoonotic pathogens [3,4].

In wild ecosystems, several enteric agents may be present, including rotaviruses. Belonging to the family *Reoviridae*, genus *Rotavirus* is of paramount importance. Rotaviruses are icosahedral, a genome with 11 double-stranded RNA segments, no lipoprotein envelope, and are classified in nine groups (A-I), based on the antigenicity of the VP6 protein. Accordingly, the groups A, D, F, and G have been reported in avian species, with clinical manifestation or no manifestation. However, the circulation in wild birds is scarce in the Amazon region [5,6,7].

Picobirnavirus (PBV) belongs to the order *Diplornavirales*, family *Picobirnaviridae*, and genus *Picobirnavirus* [8,9]. Picobirnaviruses have icosahedral symmetry, no lipoprotein envelope, and genetic material consisting of two segments of double-stranded RNA [8]. The segment 1 encodes for two proteins, one whose function is still unknown, and another one involves the capsid protein. The segment 2 codifies for RNA-dependent RNA polymerase (RdRp), and allows the classification of PBV in Genogroup I (G-I), and Genogroup II (G-II), which have been reported in several species of animals, mainly in birds [8,10]. Regarding to wild birds, Masachessi et al. [11] have previously detected the circulation of PBV in rhea, pheasant, pelican, Chinese goose, and darwin-nandu (*Rhea pennata* or *Pterocnemia pennata*) in Argentina, however the occurrence of this virus in other wild and exotic birds species is still unknown.

In this context, due to the limited knowledge on the epidemiology of these viruses and their impact in Brazilian wildlife, the present study aimed to report the circulation of RVA, RVD, RVF, and PBV in wild and exotic birds from Brazilian Amazon.

## Methods

### Ethics statement

The Biodiversity Authorization and Information System (SISBIO) of the Chico Mendes Institute for Biodiversity Conservation (ICMBio), Ministry of the Environment under No. 67300, and the Ethics Committee on the Use of Animals of Evandro Chagas Institute (CEUA/IEC) under No. 03/2019 approved this research.

All of the specimens were manipulated in Biosafety Level 3 (BSL3) laboratory of Evandro Chagas Institute, owing to the fact of the wild and exotic avian are possible reservoirs for pathogens.

### Study area and collection of clinical specimens

From January 2018 to June 2019, 23 fecal samples were collected from different species of wild birds, including two samples from toucan and *Chestnut-eared aracari* (*Ramphastidae)*, three from *Rufous-bellied thrush*, *Pale-breasted thrush*, *Turdus* sp. (*Turdidae*), four from *Spectacled owl*, *Tropical screech-owl*, *Spectacled owl*, *Striped owl* (*Strigidae*), four from *Savanna hawk*, *Roadside hawk*, *Savanna hawk*, *Roadside hawk* (*Accipitridae*), one from *Southern Caracara* (*Falconidae*), one from canary (*Fringillidae*), two from *Chestnut-bellied seed-finch*, *Sporophila sp*. (*Thraupidae*), one from cockatiel (*Cacatuidae*) and five samples from orange-winged parrot, yellow-crowned parrot, orange-winged parrot, red-browed parrot, orange-winged parrot (*Psittacidae*). All of the species were hospitalized in the Veterinarian Hospital of the Federal University of Pará (HOVET-UFPA), in Castanhal, recognized as a reference in clinical care for wild animals in Pará state.

All of the avian in this study was rescued from different cities from anthropic areas of Pará state, and they had fractures, diarrhea, dehydration, and debility of respiratory, gastrointestinal, and cardiovascular systems. The avian were treated at the HOVET-UFPA, and sent to the Municipal Health Secretary (SESMA).

The diarrheic (n = 19), and non-diarrheic (n = 4) animals came from eight different cities of Pará: Belém (n = 3), Benevides (n = 2), Capanema (n = 2), Capitão-Poço (n = 1), Castanhal (n = 10), Inhangapi (n = 1), Paragominas (n = 2), and Santa Izabel (n = 2). The fecal specimens were collected fresh, immediately after excretion, using sterile plastic bags, avoiding contact with contaminating materials. The samples were labeled, stored in sterile, sealed, and refrigerated in an universal collector tube at -20°C. Afterward, they were sent to Evandro Chagas Institute.

### Laboratory methodology

Fecal suspensions were prepared at 10% (w/v) in phosphate buffered saline (PBS 1X pH 7.4). The viral genome was extracted using silica glass powder [12], and the viral enrichment was done added 600μl of fecal suspension to 800μl of L6 buffer. Polyacrylamide gel electrophoresis (PAGE), and silver staining were applied in all specimens to detect rotavirus and picobirnavirus, according to Pereira et al. [13].

The detection of RVA was performed for all 23 samples with RT-qPCR targeting the NSP3 gene as described by Zeng et al. [14], using Taqman probe. The samples with Cycle Threshold (CT) < 40 were considered positive.

The positive samples from RT-qPCR were amplified by RT-PCR/Nested reaction. Molecular characterization of RV was done using several primers sets (**Table 1**) including: a) Gen-NSP3-F/R primers for the RVA NSP3 Gene [15]. b) N-VP7F1/R1 and N-VP7F2/R2 for the RVA VP7 gene [16]. c) N-VP4F1/R1 and N-VP4F2/R2 for the RVA VP4 gene [16]. d) SEG 10-C-S (+)/(-) for the RVA NSP4 gene [17]. e) RD6F/R, RF6F/R and RG6F/R for the RVD [18], RVF [18] and RVG [19], respectively, amplifying the VP6 genes.

**Table 1:** Primer sets used to RV detection and genotyping.

NAME	GROUP	GENE	AMPLICON	METHOD
RT-qPCR(+)/ Rotavirus A(-)/ NSP3—Probe	RVA	NSP3	140 base pairs	Real-Time [14]
Gen-NSP3-F/R	RVA	NSP3	1078 base pairs	RT-PCR [15]
N-VP7F1/R1	RVA	VP7	333 base pairs	RT-PCR [16]
N-VP7F2/R2	RVA	VP7	193 base pairs	NESTED PCR [16]
N-VP4F1/R1	RVA	VP4	257 base pairs	RT-PCR [16]
N-VP4F2/R2	RVA	VP4	214 base pairs	NESTED-PCR [16]
SEG 10-C-S (+)/(-)	RVA	NSP4	642 base pairs	RT-PCR [17]
RD6F/R	RVD	VP6	742 base pairs	RT-PCR [18]
RF6F/R	RVF	VP6	846 base pairs	RT-PCR [19]
RG6F/R	RVG	VP6	876 base pairs	RT-PCR [19]

For the detection of picobirnavirus, all of 23 samples were performed with the primers pairs PicoB25F/B43R for the Genogroup I, and PicoB23F/24R for the Genogroup II [20].

In order to characterize Genogroup I, a first round of Nested PCR was performed on the positive samples with the PBV1.2 FP/RP primers pair according to the protocol described by Malik et al. [21] for both Genogroups. A second round of Nested PCR was used the pair of forward Malik-2-FP primers (5’-TGG GWT GWT GGC GWG GAC ARG ARGG-3 ’), and Malik-2-RP reverse (5’-YSC AYT ACA TCC AC-3 ’TCC), which amplifies a partial 580 bp RdRp fragment only for the Genogroup I (designed for this study).

Briefly, in the first RT-PCR/Nested step, a mix with 4μL of extracted RNA and 1μL of each primer pair 20mM wase denatured at 97°C for 5 min in a thermocycler, followed by 5 min in an ice bath. Then, the cDNA was obtained by adding 2μL of dNTP, 2.5μL of 5X buffer, 0.75μL of MgCl, 0.5μL of Reverse Transcriptase enzyme, and 13.25μL of RNAse/DNAse free H_2_O, completing a final volume of 25μL. The reaction was incubated at 42°C for 1h.

Therefore, the cDNA was amplified to a final volume of 50μL, added to the 25μL of cDNA, 3μL of dNTP mix, 2.5μL of 5X buffer, 0.75μL of MgCl, 0.25μL of Taq Polymerase (Invitrogen), and 18.50μL of RNAse/DNAse free H_2_O. All the PCR cycle conditions are available on the **Table 2**. The Sanger di-deoxy method of nucleotide sequencing [22] was performed using the same primer pair used in the RT-PCR, and for all amplicons obtained. The sequences were aligned, and edited using Geneious software v.10.0.6 [23]. Afterward they were compared with other sequences deposited in GenBank through the Basic Local Alignment Search Tool (BLAST). Phylogenetic trees were constructed by MEGA V.6 program [24], based on Kimura parameters [25] using the non-parametric reliability test with bootstrap of 1000 replicas.

**Table 2:** RT-PCR/Nested conditions for amplification for rotavirus and picobirnavirus.

CONDITION	VIRUS				
	RVA NSP3	RVA VP7/VP4	RVA NSP4	RVD/RVF/RVG VP6	PBV RdRp
INITIAL DENATURATION/TIME	94°/3min	94°/3min	94°/3min	93°/3min	94°/3min
CICLE	35X	35X	35X	35X	35X
DENATURATION/TIME	94°/45sec	94°/45sec	94°/45sec	93°/1min	94°/45sec
HIBRIDIZATION/TIME	45°/30sec	45°/30sec	45°/30sec	55°/1min	45°/30sec
EXTENTION/ TIME	72°/1min	72°/1min	72°/1min	72°/1min	72°/1min
FINAL CICLE/TIME	72°/1min	72°/1min	72°/1min	68°/7min	72°/1min

### Statistic test

We performed a descriptive statistic test for continuous variables of animal ages, detections for RV, PBV, and diarrhea sign from absolute, and relative frequency test. For categorical variables, Chi-square or Fisher Test was executed when appropriated on SPSS v.12.0 software with p value ≤0.05.

## Results and discussion

**Fig 1:**
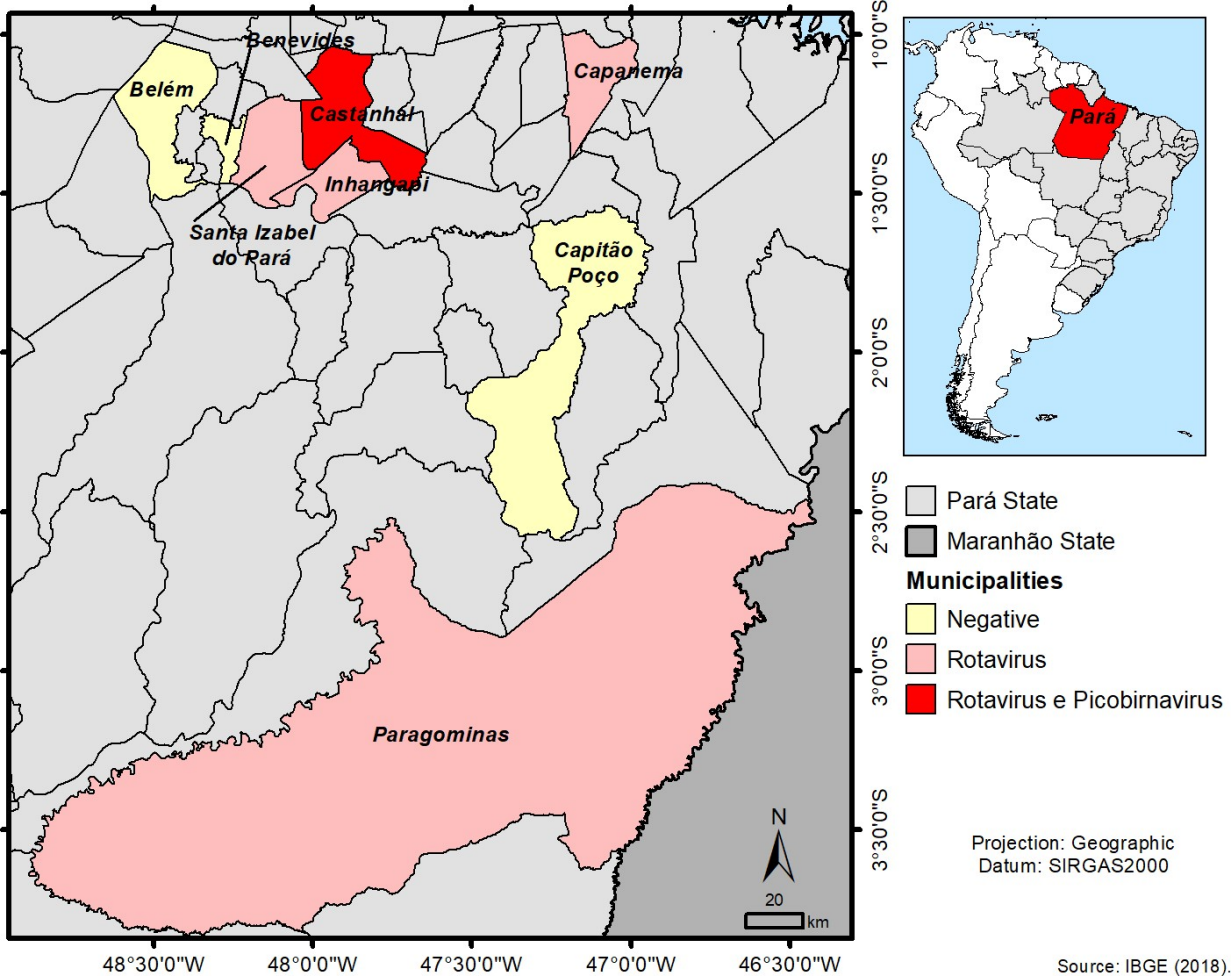
Geographic distribution of positive cases of RV and PBV in wild and exotic birds in the state of Pará. All data were built with public demographic data available on IBGE (Brazilian Institute of Geography and Statistics) with SIRGAS2000 (Geocentric Reference System for the Americas) obtaining coordinates from Pará state, Brazil and It can be found in https://www.ibge.gov.br/geociencias/organizacao-do-territorio/malhas-territoriais/15774-malhas.html?=&t=downloads.

All samples were tested by PAGE, RT-qPCR, and RT-PCR/Nested for RVA (NSP3, VP7, VP4, and NSP4 genes), RVD (VP6 gene), RVF (VP6 gene), RVG (VP6 gene), and PBV (RdRp gene). The analysis by PAGE showed negativity for 23 samples, and there was no electrophoretic migration characteristic of RV and PBV. However, RT-qPCR was positive in five of eight cities for RVA, detected in 39.1% (9/23) of avian fecal specimens from Castanhal, Capanema, Inhapagi, Paragominas, and Santa Izabel (**Table 3**). No case was registered in Belém, Benevides, and Capitão-Poço. On the other hand, Castanhal had the highest frequency, 44.5% (4/9) of the cases, and exhibited positivity for both RV and PBV (**Fig 1**).

**Table 3:** Positive RV and PBV samples from animals hospitalized at the Veterinary Hospital of the Federal University of Pará, Brazil.

FAMILY/ SPECIES	BRAZILIAN/ENGLISH POPULAR NAME	REASON FOR HOSPITALIZATION	CITY FROM	RT-qPCR RV	RT-PCR RV	PBV
*Turdidae*/ *Turdus rufiventris*	Sabiá-laranjeira/ Rufous-bellied Thrush	Diarrhea	Castanhal	39.1	G1/P[2]	Neg
*Turdidae*/ *Turdus sp*.	Sabiá/Thrush	Diarrhea	Paragominas	34.8	G3/**P[2]**	Neg
*Fringillidae*/ *Serinus canaries*	Canário-do-reino/ Canary	Pododermatitis, tachypnea and diarrhea	Castanhal	38.2	G3	Neg
*Thraupidae*/ *Sporophila angolensis*	Curió/ Chestnut-bellied Seed-Finch	Diarrhea	Paragominas	38.5	G6	Neg
*Psittacidae*/ *Amazona amazonica*	Papagaio-do-mangue, curica/ Orange-winged Parrot	Lameness, limb atrophy and diarrhea	Inhangapi	Neg	G3/**P[2]**	Neg
*Accipitridae*/ *Heterospizias meridionalis*	Gavião-caboclo/ Savanna hawk	Humeral fracture and diarrhea	Castanhal	38.5	T2/G3/ **P[2]**	Neg
*Psittacidae*/ *Amazona amazonica*	Papagaio-do-mangue, curica/ Orange-winged Parrot	Respiratory, cardiovascular and ocular systems injury	Santa Izabel	33.0	RVA/ RVD/ RVF	Neg
*Ramphastidae*/ *Ramphastus sp*	Tucano/Toucan	Diarrhea	Castanhal	38.0	G3/**P[2]**	GI
*Cacatuidae*/ *Nymphicus hollandicus*	Calopsita/Cockatiel	Apathy, diarrhea and depression	Capanema	39.0	G3	Neg

Neg = Negative.

The frequency of diarrhea sign was 82.6% (19/23) with a higher frequency of 60.9% in adult animals (14/23). The frequency of positivity for rotavirus was found in 9/23 birds (39.1%), and 3/9 (33.3%) were in adult animals with 8/9 (88.8%) diarrheal signs. Refering to picobirnavirus 1/23 (4.3%) sample was positive. It belongs a non-adult that presented diarrheal sign. Therefore, due to a low number of samples for analysis in this study, this fact contributed for no significance for p ≤0.05 between rotavirus, and picobirnavirus detection, diarrhea sing, and animal age, as shown in (**Table 4)**.

**Table 4:** Distribution of animals’ age, diarrhea sign, rotavirus and picobirnavirus detection in birds attended in the Veterinarian Hospital of Castanhal, 2018–2019.

Variable	n	%	RV P value	PBV P value
Adult animal				
Yes	14	60.9	0.643	0.391
No	9	39.1	1.000	0.639
Diarrhea sign				
Present	19	82.6		
Absent	4	17.4		
Rotavirus detection				
Yes	6	26.1		
No	17	73.9		
Picobirnavirus detection				
Yes	1	4.3		
No	22	95.7		
**Total**	**23**	**100.0**		

Notably, the results are contrasting with the findings of Guerreiro et al. [26] that reported negative results for the 23 fecal samples of migratory birds for rotavirus, and PBV, using the same primers of this study. It is worth mentioning that these animals did not present clinical signs differently from the signs presented in the animals of present study, and it could be an indication of lower viral load presented by these animals. In addition, the low sensitivity of the PAGE was recorded, and may be justified due to the low viral load excreted by birds, corroborating the data from Masachessi et al. [11], Guerreiro et al. [26], Fregolente et al. [27], and Barros et al. [28], which displayed no electrophoretic profile in the positive samples. In relation to PBV, the primers pair used by Rosen et al. [20] is limited generating a short amplicon of 200 bp for Genogroup I, and ~300 bp for Genogroup II, which can lead a negative result. This is the reason in the present study we used the Nested PCR by Malik et al. [21].

The higher CT-values in RT-qPCR ranged from 33 to 39.1 (mean = 37.38), hence indicating the presence of low viral load of RVA in wild specimens. Remarkably, Barros et al. [28] demonstrated that RT-qPCR assay is an efficient tool to detect RVA in specimens with low viral load. In this study, it was further possible to perform detection and genotyping for NSP3, VP7 and VP4 genes of RVA, with greater recovery of RVA sequences. This occurred probably due to the use of the nucleic acid extraction method (silica glass powder) described by Boom et al. [12], in contrast to Barros et al. [28] who used TRIzol, and characterized 1.25% (8/648) of the samples only for the VP4 gene. In fact, TRIzol has the capacity to extract RNA with high integrity. However, chloroform residues can consume the RT-qPCR reagents, and may cause amplification failure, hence justifying the low rate of molecular characterization reported by Barros et al. [28].

Our specimens had low load viral, and the short nucleotide sequences obtained of the VP7 (~193bp), and VP4 (~214bp) genes could not characterize these genes. Even with the enrichment viral step, and the sequences analyses just shown the different clades of reported genotypes, we could describe the prevalence of G3 equine-like (7/9), G1 (1/9), G6 (1/9), and **P[2]** (5/9) genotypes among the avian. Thus, the nucleotide sequences in this study showed more similarity to the human strains owing to limited nucleotide sequences of wild birds origin in databases [29]. The genotypes already reported circulating in birds are T4 and T8 for NSP3 gene [17,30–33], **G5** [32], **G6** [34], **G7** [35], **G8** [36], **G10** [34], **G11** [33], **G17** [33], **G18** [36], **G19** [36], **G22** [37], **G23** [30] for VP7 gene [31,34–38], and **P[1]** [37], **P[17]** [35], **P[30]** [17], **P[31]** [35], **P[35]** [28] **and P[37]** [29], **P[38]** [28] for VP4 gene [18,30,41,37,39].

**Fig 2:**
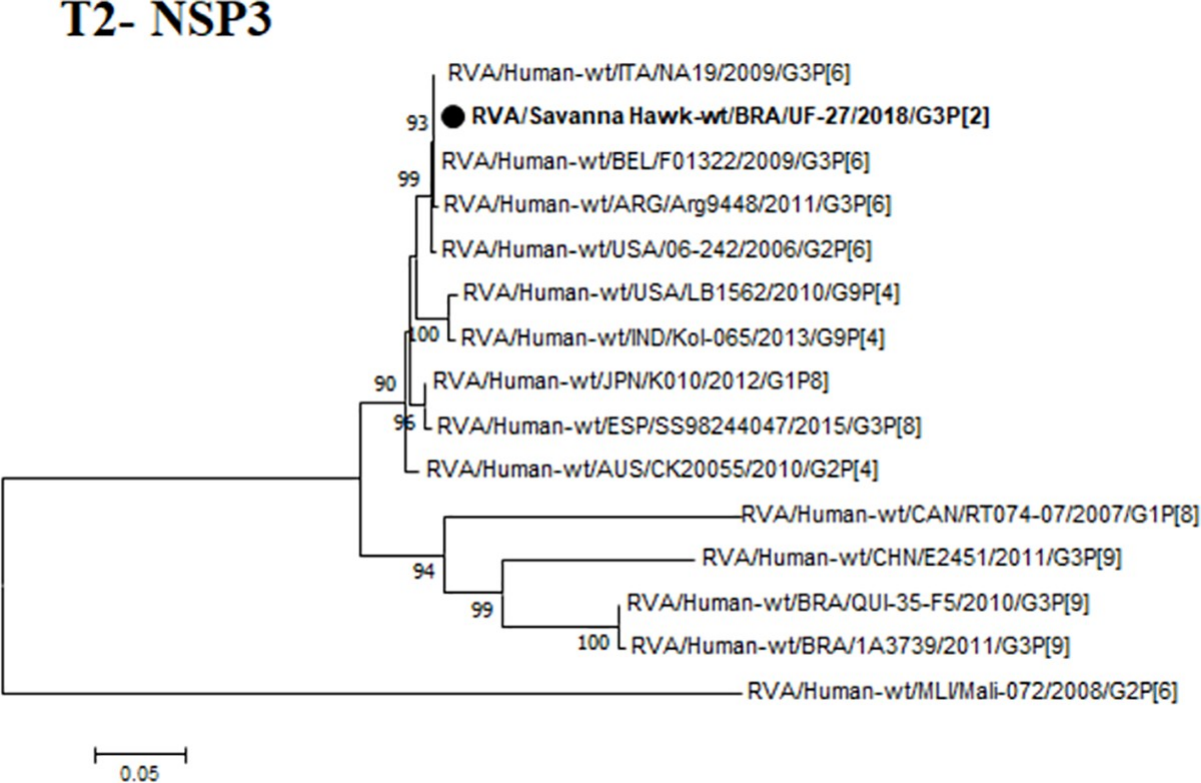
Phylogenetic tree based on the alignment of RVA NSP3 gene sequences. The sequence of this study is represents in bold, and the black color depicts the birds. The numbers next to the nodes indicate bootstrap values > 70.

In this study, one sample of nine was generated with specific amplicon of 1078 bp for the T2 genotype of the NSP3 gene in an adult Savanna Hawk (*Heterospizias meridionalis*) of undeterminated gender, which belonged to the T2 genotype of RVA, revealing 100% nucleotide homology with a human strain detected in Belgium in 2009 (JF460831), as shown in **Fig 2**. Importantly, this is the first report involving the circulation of T2 genotype in wild birds. Since this sample belongs to a rescue bird next to BR-316 road and we suggest the emergence of the Genotype T2 in the Amazon region, evidencing a possible circulation in wild birdlife.

**Fig 3:**
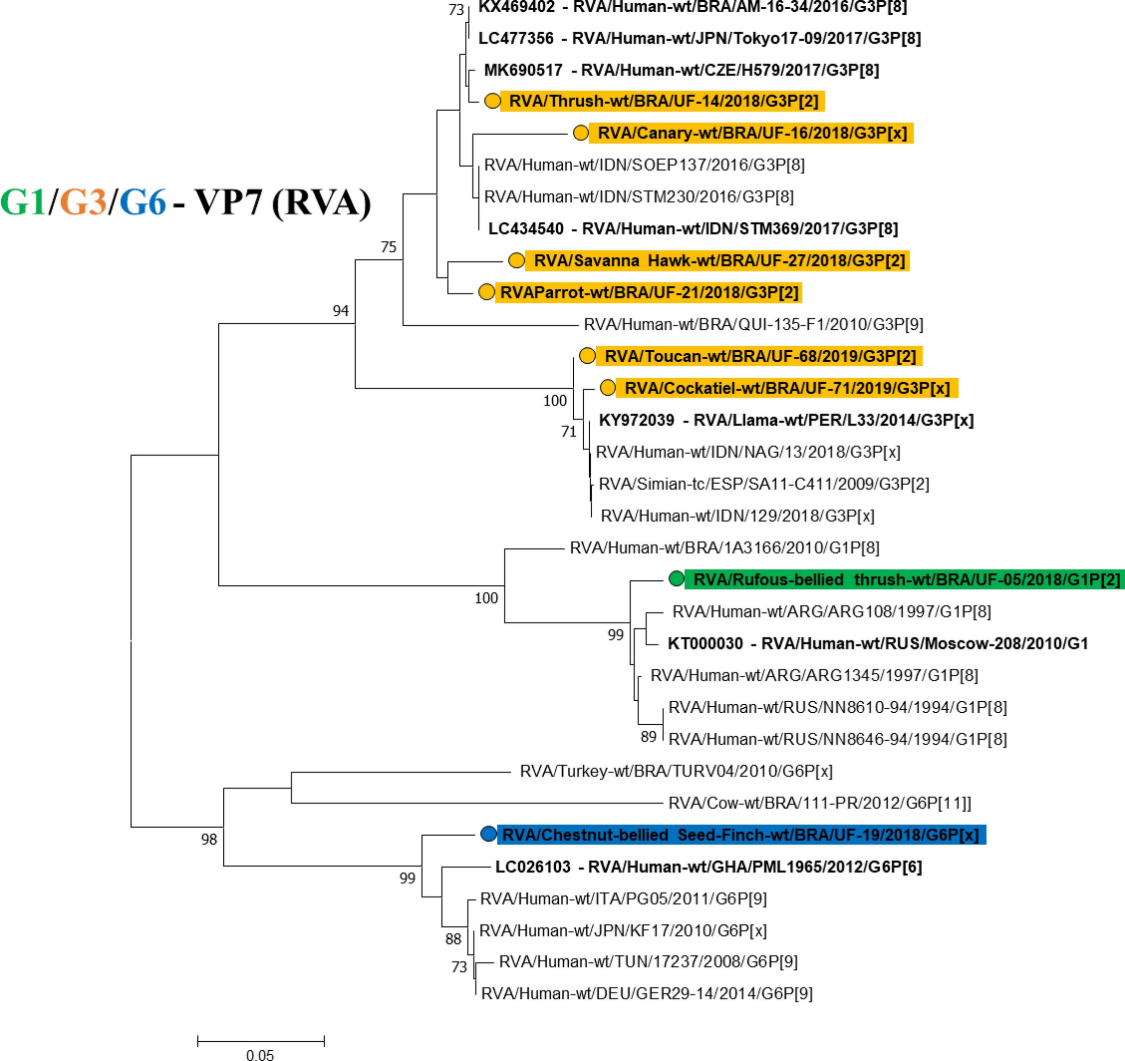
Phylogenetic tree based on RVA VP7 gene sequence alignment. The sequences of this study are represent in different colors. The orange color depicts the G3 genotype, the green color depicts the G1 genotype, and the blue color depicts the G6 genotype. The numbers next to the nodes indicate bootstrap values > 70.

Regarding the VP7 gene, it was possible to obtain eight of nine sequences of the samples, generating a 193 bp amplicon (**Fig 3**) with G3 equine-like prevalence (6/9), and the same hawk sample grouped in the unusual G3 equine-like genotype with proximity to an Orange-winged Parrot (*Amazona amazonica*) sample which also belong to this genotype. This sample was collected in 2018, and grouped in the same clade.

The sample from canary (*Serinus canaria*), also collected in 2018 was grouped in a different clade, reporting G3 equine-like strain [40]. The canary, and the Savanna Hawk were obtained from Castanhal, and the Orange-winged Parrot from Inhangapi. These birds showed clinical signs of diarrhea.

These findings emphasize the importance of continuous monitoring of RVA in virtue of the previous report of the equine-like G3 genotype in the Amazon Region [41], originated from the selective pressure of zoonotic strains, and due to the possible presence in different ecological niches mainly in anthropic areas. The diarrhea sample from a thrush (*Turdus sp*.) came from Paragominas formed a clade separately from the other samples of the G3 genotype. The other two diarrheic samples belonging to a toucan (*Ramphastus sp*.), and a cockatiel (*Nymphicus hollandicus*) were obtained from Castanhal and Capanema, respectively. These specimens were grouped in the same clade [42]. This genotype is considered the most virulent, justifying the diarrheal condition evidenced by the animals grouped in the G3 genotype [41].

The RVA genotype G3 is considered the third most common genotype due to a larger spectrum of hosts, and a greater potential for interspecies transmission in comparison to the human genotypes G1, G2, and G4. This genotype is circulating in wild animals, as well as in others, such as cattle, canines, equines, swine, leporids, ovine, camelids, rodents, felines, simians, bats, and also in humans [29]. The present study is the first to report the circulation of G3 genotype in wild birds, and exotic species, and scientific evidence indicates the possible interspecies transmission or exposure of these animals to the same source of contamination.

In 2017, Bezerra et al. [43] reported the circulation of the G3 genotype in samples from quilombolas of the Amazon Region, which had similarity with G3 from animal origin (simian, bats, llama, horse, and alpaca). Nevertheless, further studies on the genotypic constellation of the RV genomic segments on the specimens evaluated are needed in order to obtain data regarding their distribution, and the possible natural reservoirs of the G3 genotype in wild fauna.

One diarrheic sample from Rufous-bellied Thrush (*Turdus rufiventris*) collected in Castanhal reported G1 genotype, which has been reported in humans [44], bears [45], sheep, cattle, llamas [46], and pigs [47]. In addition, one diarrheic sample from Chestnut-bellied Seed-Finch (*Sporophila angolensis*) from Paragominas showed the human G6 genotype, and it has been reported in birds (this genotype was related in turkey farms, and there is no reports on wild birds) [39], cattle [47], sheep [48], pigs [49], horses [50], antelopes [51], leporids [52], doe [53], and human [54].

The birds to the order *Passeriformes* (seed-finch and thrush) represent the most illegally marketed group, corresponding to 90% of the avian traffic, due to their beautiful singing. In turn, *Psittaciformes* (parrot) represent 6%, due to their colored feathers, and other genera of birds correspond to 4% [55]. In this study, the human genotypes circulating in seed-finch, and thrush can be justified owing to human contact arising from the illegal commercialization of these birds, characterizing the first record in worldwide of genotypes G1 and G6 in wild birds.

**Fig 4:**
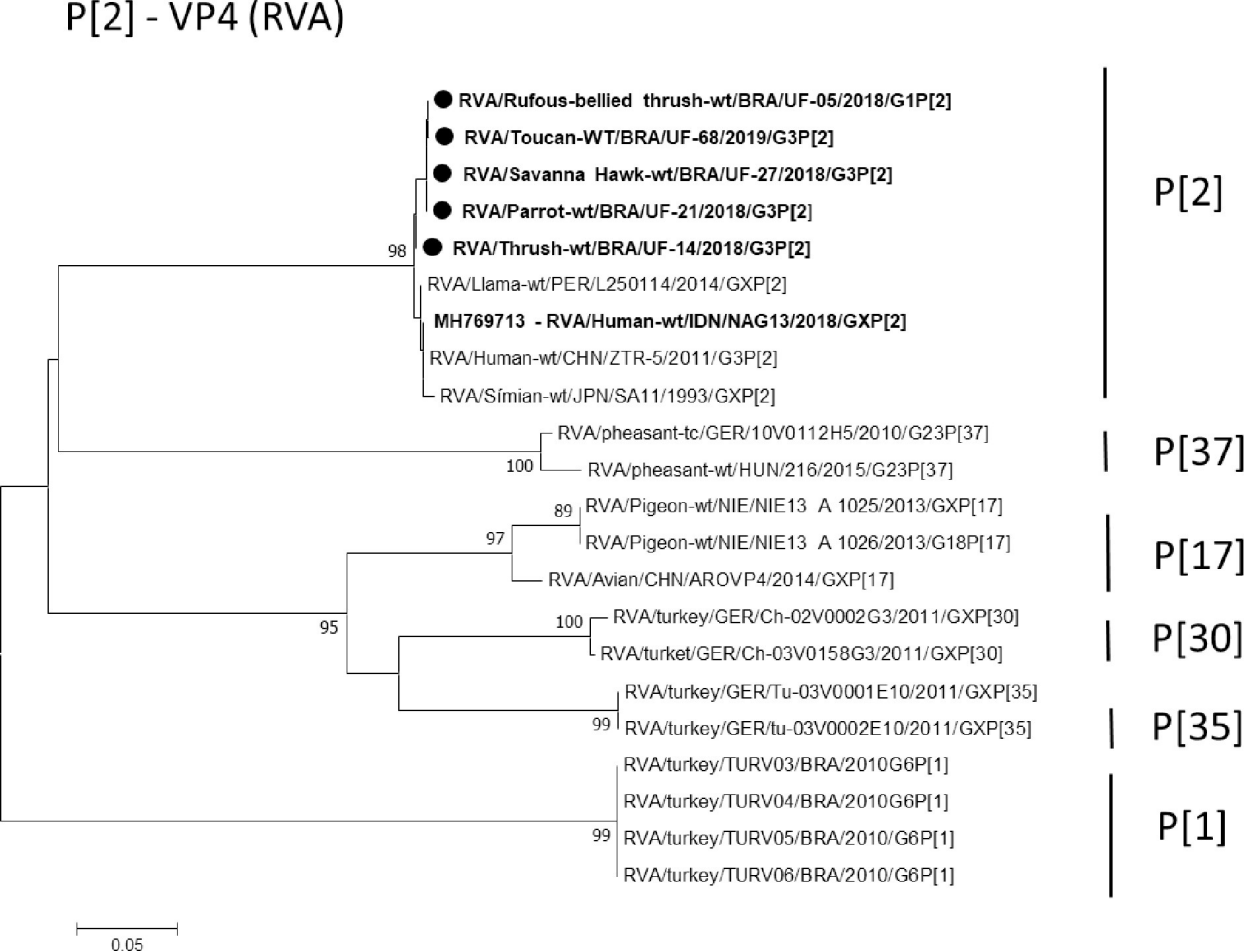
Phylogenetic tree based on the sequence alignment of the avian RVA VP4 gene. The sequence of this study is represents in bold, and the black color depicts the birds. The numbers next to the nodes indicate bootstrap values > 70.

Regarding to VP4 gene, genotype **P[2]** is an uncommon one, rarely reported in the literature, which has been recently studied. However, it has been found in humans, simians [56,57], llamas, and alpacas [42]. In this study, it was possible to obtain five of the nine sequences of the samples, producing an amplicon with 214 bp. All the samples grouped in the same clade showed distance from other samples detected worldwide that presented the **P[2]** genotype, as shown in **Fig 4**. These specimens came from one thrush (*Turdus sp*.) from Paragominas, one Orange-winged Parrot (*Amazona amazonica*) from Inhangapi, one toucan (*Rhamphastus sp*.), one Rufous-bellied Thrush (*Turdus rufiventris*), and one Savanna Hawk (*Heterospizias meridionalis*) from Castanhal.

Therefore, it is the first report of the circulation of genotype **P[2]** in Brazil. Recently, Rojas et al. [42] reported the unusual genotype **P[2]** circulating in llamas, alpacas, and humans in Peru, thus revealing the zoonotic potential associated with the circulation of genotypes G1, and G3 in these animals. The findings suggest the close interaction of humans, and wild animals that can result in the breaking of the barrier between species, resulting in the adaptation of RV to new hosts.

These data corroborate with Asano et al. [39], and Da Silva et al. [58] who reported bovine, and swine genotypes in birds, emphasizing the interspecies transmission of RVA involved in the wild cycle on Amazon. Previous study of Luchs and Timenetsky [29] showed the prevalence of genotype G3 in wildlife. However, noted birds represent 80% of animal species illegally marketed in Brazil. This practice imposes a risk to biodiversity, and affects the health of animals, which are often in precarious situations such as malnutrition, abrasions, immune weakness, overcrowding in cages, absence of hygienic space, and which may be exposed to different etiological agents. Wild and exotic birds are considered potential reservoirs for zoonotic diseases, human and environmental health can be influenced, unbalancing the biological cycle of several pathogens, justifying the presence of unusual genotypes in the specimens under study [55,59].

However, due to high capacity of rearrangement of RV, and PBV more studies must be conducted with all genomics segments. In addition, more samples of wild animals need to be collected to report molecular datas, contributing to understand the transmission pattern between animals and humans as well as analyze the prevalence of strains in different populations correlating the viruses with environmental degradation.

**Fig 5:**
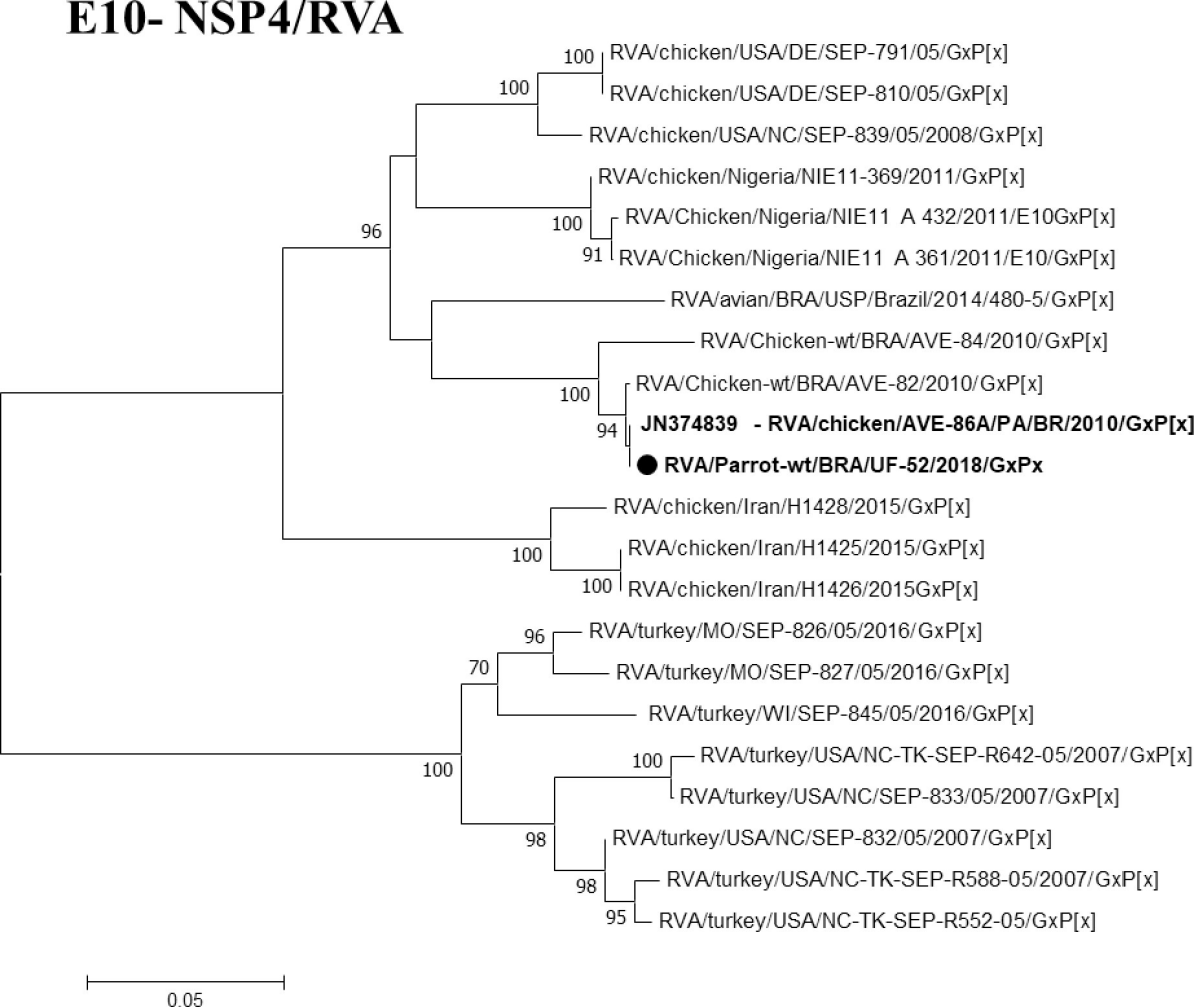
Phylogenetic tree based on the alignment of avian RVA NSP4 gene sequences. The sequence of this study is represents in bold, and the black color depicts the birds. The numbers next to the nodes indicate bootstrap values > 70.

**Fig 6:**
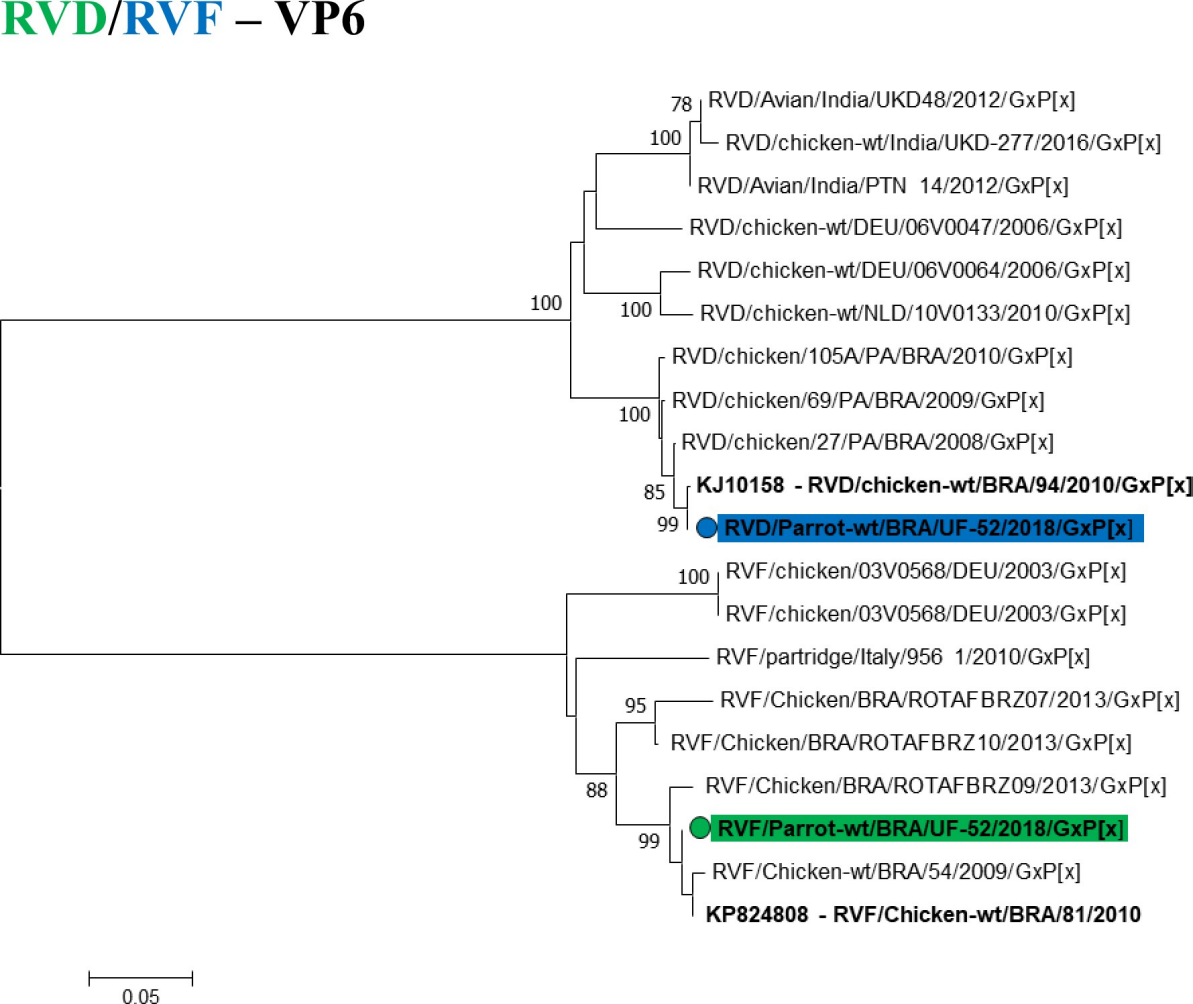
Phylogenetic tree based on the sequence alignment of the VP6 gene of RVD and avian RVF. The sequences of this study are represent in different colors. The blue color depicts the RVD, the green color depicts the RVF. The numbers next to the nodes indicate bootstrap values > 70.

Regarding to avian rotaviruses (RVA, RVD, RVF and RVG), one sample was amplified simultaneously for RVA, RVD, and RVF (1/23). They produced amplicons of 642 bp (NSP4) (**Fig 5**), 742 bp, and 846 bp for the VP6 gene (**Fig 6**). The sample was obtained from an Orange-winged Parrot (*Amazona amazonica*) from Santa Izabel, which showed no clinical signs of diarrhea, however, manifested deficiency of cardiovascular, ocular, and upper respiratory systems, rendering the bird susceptible to viral infections.

Barros et al. [27] reported the presence of RVA by RT-qPCR in 23.6% of poultry, and wild birds circulating in the Amazon Brazilian biome. Bezerra et al. [18], Da Silva et al. [58], and Mascarenhas et al. [19] detected RVD, RVA, RVF, and RVG in broiler chicken in the same cities explored in this study (Belém, Ananindeua, Inhangapi, Benevides, Castanhal, and Santa Izabel). In this study, the parrot specimen reported 100% similarity with the reference strains for RVA (JN374839), RVD (KJ101587), and RVF (KP824808) circulating in the Amazon Region. No positivity was recorded for RVG.

In Amazon Region, Luz et al. [60], and Guerreiro et al. [26] investigated rotavirus A, and D in wild captive, and migration birds, respectively. Accordingly, the researchers did not observe the circulation of RV and PBV after using the same primers of this study. Contrarily, Guerreiro et al. [26] designed a primer targeting the VP7 gene, and obtained 1/23 positivity for avian RVA.

**Fig 7:**
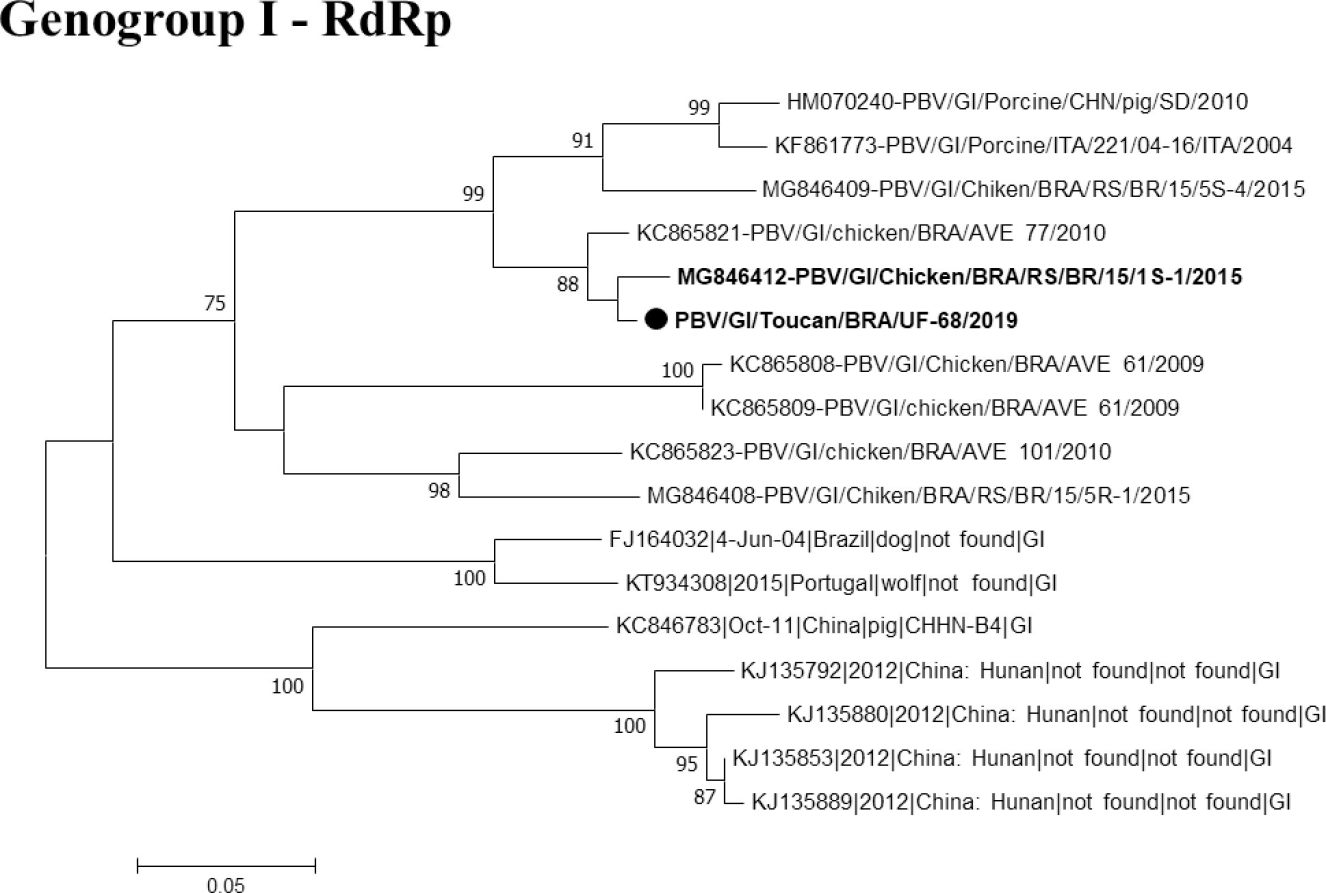
Phylogenetic tree based on sequence alignment of the PBV RdRp gene. The sequence of this study is represents in bold, and the black color depicts the birds. The numbers next to the nodes indicate bootstrap values > 70.

Regarding to PBV, all the 23 samples were tested for both GG-I, and GG-II, and one (1/23) amplified for the RdRp gene yielded an amplicon of 580 bp. This specimen came from a toucan (*Ramphastus sp*) from Castanhal with sign of diarrhea, and showed coinfection for RV. The sample was grouped in Genogroup I of PBV with the *Chicken picobirnavirus* strain, showing 97% nucleotide similarity with the reference strain MG846412, which was reported in Rio Grande do Sul in 2015 in birds [33], and 98% with KC865821 circulating in the Amazon Region in 2010 [35], as shown in **Fig 7**. Therefore, this is the first report of RdRp PBV gene in wild birds in Brazil circulating in toucans from the Amazon region.

The absence of diarrheal symptoms resulting from PBV infections is reported in several hosts [10], which can be one of the factors interfering in the diagnosis, considering that the amount of viral particles excreted in the feces is not detectable by PAGE. Thus, due to the low viral load, no positive signal in PAGE was observed in this study, thereby suggesting that PBVs were not the primary agent for the manifestation of diarrheal conditions, since the animal presented coinfection for rotavirus.

However, it is suggested that the diarrheic animals that were not positive for PBV could be affected by other enteric agents (viral, fungal, bacterial, protozoan or helminthic), and likely have triggered diarrheal conditions due to stress caused by physiological disorders, such as fractures, myiasis, tachycardia, tachypnea, and other injuries. For those animals positive for RV with no signs of diarrhea, we suggested that the low viral load in the samples may explain the absence of any manifested symptoms (observed in the CT’s obtained in the RT-qPCR).

The frequency of cases in Castanhal was higher than other cities, problably due to Castanhal has more avian samples to this study. However, we also can consider that Menes and Simonian [61] interviewed street market merchants from Bragança, Cametá, Capanema, Castanhal, Paragominas, Santarém and Tucuruí (city of state of Pará) regarding to clandestine commercialization of wild animals, and the data showed that Castanhal exhibited the highest commercialization of these species. Such information could corroborate with our study considering the high prevalence of RV and PBV in Castanhal, and demonstrate that this illegal activity could induce interspecies transmission, interfering in the transmission of viral agents including those pathogens between ecosystems, and their genetic diversity.

Thus, Barros et al. [28], Guerreiro et al. [26], Luz et al. [60], Da Silva et al. [58], Mascarenhas et al. [19], and Bezerra et al. [18] corroborate for the present study. The literature reports suggest that the Metropolitan regions of Belém and Northeast of the state of Pará concentrate the highest rates of anthropic pressure in the Legal Amazon, thus unveiling that the heterogeneity of RV and PBV in birds is co-circulating in urban, rural, and wild ecosystems. The survey studies in anthropic areas are important to investigate due to proximity with enterprises and rural habitat. In this area has a variety of host for different pathogens that could circulate in urban populations. Although the present study had low population (23 samples), this investigation is important to scarce epidemiologic data in wild population. Thus, these results shed new light on the potential influence of avian in RV’s and PBV’s epidemiology, and their important role as potential reservoirs of broad range of genotypes, usually considered typical solely from domestic animals, and humans cannot be dismissed.

## Conclusion

Epidemiological data on the dynamics of enteric viruses in wildlife of the Amazon region are still scarce. Although the short amplicon obtained, this study is a pioneer in reporting the prevalence of G3 equine-like, G6, G1 (VP7 gene), **P[2]** (VP4 gene), T2 (NSP3 gene) genotypes of RVA, avian rotavirus (A, D, F), and PBV in wild and exotic avian circulating in the Amazonian urban habitat.

Therefore, additional evidence in wild birds and exotic species is required as genotype constellation in order to provide a comprehensive understanding of the biological cycle of rotavirus, and picobirnavirus in these animals. Further, the interactions with avian species, dispersal among human and other animals, the identification of the predominant strains to determine the role of these birds in the epidemiology of the disease, and to develop prophylactic measures are of utmost importance.
